# Artificial Intelligence in Dermoscopy: Enhancing Diagnosis to Distinguish Benign and Malignant Skin Lesions

**DOI:** 10.7759/cureus.54656

**Published:** 2024-02-21

**Authors:** Shreya Reddy, Avneet Shaheed, Rakesh Patel

**Affiliations:** 1 Biomedical Sciences, Creighton University, Omaha, USA; 2 Pathology, University of Illinois College of Medicine, Chicago, USA; 3 Internal Medicine, Quillen College of Medicine, East Tennessee State University, Johnson City, USA

**Keywords:** dermoscopy image analysis, benign lesions, malignant lesions, skin lesions, artificial intelligence (ai)

## Abstract

This study presents an innovative application of artificial intelligence (AI) in distinguishing dermoscopy images depicting individuals with benign and malignant skin lesions. Leveraging the collaborative capabilities of Google's platform, the developed model exhibits remarkable efficiency in achieving accurate diagnoses. The model underwent training for a mere one hour and 33 minutes, utilizing Google's servers to render the process both cost-free and carbon-neutral. Utilizing a dataset representative of both benign and malignant cases, the AI model demonstrated commendable performance metrics. Notably, the model achieved an overall accuracy, precision, recall (sensitivity), specificity, and F1 score of 92%. These metrics underscore the model's proficiency in distinguishing between benign and malignant skin lesions. The use of Google's Collaboration platform not only expedited the training process but also exemplified a cost-effective and environmentally sustainable approach. While these findings highlight the potential of AI in dermatopathology, it is crucial to recognize the inherent limitations, including dataset representativity and variations in real-world clinical scenarios. This study contributes to the evolving landscape of AI applications in dermatologic diagnostics, showcasing a promising tool for accurate lesion classification. Further research and validation studies are recommended to enhance the model's robustness and facilitate its integration into clinical practice.

## Introduction

Skin lesions, encompassing both benign and malignant conditions, represent a pervasive and intricate facet of global dermatological health. These lesions manifest in diverse forms, ranging from innocuous benign growths to potentially life-threatening malignant neoplasms [1]. In 2017, there were almost 10 million diagnosed cases of benign skin lesions [2]. Benign skin lesions currently affect more than three million in the United States per year, and malignant skin lesions (such as melanoma) impose a significant burden of disease due to their potential for rapid progression, metastasis, and adverse outcomes if left untreated, particularly the metastasis to vital organs such as the lungs, liver, or brain [2,3]. Understanding the prevalence and formation of these lesions is crucial for contextualizing the significance of accurate diagnostic tools [1]. The global incidence of skin lesions is substantial, affecting millions of individuals across diverse demographics [4]. In the United States, it is estimated that one in five Americans will develop skin cancer (either malignant or benign) by the time they are 70 years old [5]. Skin cancers, including malignant melanoma and non-melanoma skin cancers, contribute significantly to the global burden of cancer. Current data suggest that there will be around 200,000 new cases of malignant melanoma that will be diagnosed in 2024 [5]. Additionally, benign skin lesions, such as moles, cysts, and dermatofibromas, are prevalent in a large segment of the population, adding to the overall prevalence of dermatological conditions [4]. Given the gravity of this issue, streamlining the diagnostic process for dermatologists could prove invaluable in expediting patient treatment and care.

The formation of skin lesions is a multifaceted process influenced by various genetic, environmental, and lifestyle factors [6]. Benign lesions often arise due to hyperplasia of skin cells, leading to the formation of growths such as moles or dermatofibromas [7]. These lesions are generally noncancerous and pose minimal health risks. Malignant skin lesions, on the other hand, originate from the uncontrolled growth of mutated skin cells, typically triggered by exposure to ultraviolet (UV) radiation, genetic predispositions, or other carcinogenic factors [8].

Benign skin lesions encompass a diverse array of conditions, ranging from common moles to seborrheic keratoses [9]. Benign skin lesions are typically harmless growths or abnormalities, often characterized by specific visual cues. These may include regular borders, consistent coloration, and a symmetrical structure [9]. Common examples of benign lesions encompass moles, cysts, and dermatofibromas, among others [10]. Moles, or nevi, usually result from the proliferation of melanocytes, the pigment-producing cells in the skin [9]. Seborrheic keratoses, characterized by wart-like growths, arise from the overproduction of keratinocytes, the predominant cells in the epidermis [11]. While benign, these lesions can be aesthetically bothersome or cause discomfort, prompting individuals to seek medical attention. In contrast, malignant skin lesions, primarily skin cancers, arise from the malignant transformation of skin cells [12]. Malignant lesions may display irregular borders, variegated colors, and asymmetry, reflecting the invasive nature of cancerous growth [13]. Other ominous signs include changes in size, shape, or elevation of preexisting moles [14]. The most aggressive form, malignant melanoma, develops in melanocytes and can metastasize rapidly, posing a substantial threat to health [15]. Non-melanoma skin cancers, including basal cell carcinoma and squamous cell carcinoma, typically arise from the basal and squamous cells of the epidermis, respectively [16]. UV radiation from sunlight is a major contributor to the mutational processes leading to these cancers [7].

Accurate diagnosis and classification of these lesions are critical for timely intervention and effective patient management. In diagnostic dermatology, one of the prevailing challenges is the time-consuming nature of traditional diagnostic methods, which can lead to delays in treatment initiation and subsequent patient management. Moreover, the subjective interpretation of dermatological findings among clinicians may introduce variability in diagnosis, impacting both accuracy and consistency. These issues underscore the pressing need for innovative solutions, such as artificial intelligence (AI)-based diagnostic tools, to enhance the efficiency, accuracy, and accessibility of dermatological diagnostics. In this context, the development of advanced diagnostic tools, such as AI models, holds promise for enhancing precision and efficiency in dermatopathology [17]. While it's true that capturing high-quality photos for AI analysis may add time to the dermatological examination process, the investment in time is outweighed by the potential benefits of improved diagnostic accuracy and treatment outcomes. AI has the potential to analyze images of skin lesions to provide automated diagnosis or assist dermatologists in their decision-making process. These systems can identify patterns and features indicative of various skin conditions, including skin cancer, psoriasis, eczema, and acne. AI methods have already been incorporated into the practice of dermatology. AI algorithms can analyze clinical and genetic data to develop personalized treatment plans for patients with dermatological conditions. By integrating patient-specific factors such as genetic predisposition, medical history, and treatment responses, AI-powered systems can recommend tailored therapies and predict treatment outcomes with greater accuracy. Telemedicine utilizes AI algorithms for image analysis and triage, enabling patients to upload photos of skin lesions for assessment by dermatologists or AI diagnostic systems. Additionally, virtual screening and molecular modeling are powered by AI, and they are accelerating the discovery and development of novel therapeutics for dermatological conditions. By analyzing vast datasets of chemical compounds and biological targets, these algorithms can identify promising drug candidates and optimize their properties for efficacy and safety. AI-driven decision support systems can provide dermatologists with real-time insights and recommendations based on a comprehensive analysis of patient data, enabling faster and more accurate diagnosis and treatment planning. Overall, AI has the potential to streamline full skin exams in dermatology by reducing the time required for manual examination and interpretation of findings, ultimately leading to improved patient care and outcomes.

By leveraging large datasets of annotated images, AI models can learn to recognize subtle differences between benign and malignant lesions with high accuracy [12]. Our study and other studies done in the past are similar in that both studies utilize machine learning techniques for the accurate detection and diagnosis of melanoma and nevi, demonstrating the potential of AI in dermatological diagnostics [12]. Like other previous studies, our study also focuses on improving the classification of skin lesions to aid in clinical decision-making and enhance patient outcomes [12]. However, this study will be different from previous studies in that we use multiple types of benign and malignant lesions [12]. Our study aims to contribute to this imperative by presenting an AI model proficient in distinguishing between benign and malignant skin lesions, with a focus on accessible, cost-free, and environmentally sustainable training methodologies using Google's Collaboration platform.

## Materials and methods

The methodology employed in this study involved a meticulous approach to training an AI model for the discrimination of pathology slides depicting benign and malignant skin lesions. The utilization of a diverse and representative dataset, acquired from Kaggle.com, and the implementation of Google's Collaboration platform for training underscored the strategic choices made to enhance the model's efficacy. What distinguishes this specific model is its comprehensive approach to classification. In the benign category of the employed dataset, a wide spectrum of conditions is considered, ranging from nevi to papules, cysts, nodules, warts, ulcers, rashes, and hives. This broad inclusion ensures a thorough assessment of various nonmalignant skin lesions, allowing for a more nuanced and accurate diagnostic process. Furthermore, in the malignant dataset used for model training, the scope extends beyond melanomas to encompass other significant malignancies such as basal cell carcinoma and squamous cell carcinoma. By incorporating diverse types of malignant lesions, the model is equipped to recognize a broader range of cancerous growths, thus enhancing its diagnostic capabilities and clinical utility.

The dataset, a pivotal component of model training, consisted of 1,000 images. A judicious balance was maintained, with 500 pathology slides illustrating benign skin lesions and an equivalent number depicting malignant lesions. This balanced distribution ensured that the model was exposed to a comprehensive range of visual patterns associated with both benign and malignant conditions. The dataset was sourced from Kaggle.com, a prominent platform for hosting publicly available datasets, offering diversity and relevance to real-world scenarios in dermatopathology. To ensure robust model evaluation and generalizability, the dataset underwent a random split into three subsets: training, validation, and testing. Notably, 80% of the data were allocated to the training set, allowing the model to learn intricate patterns and features. The validation set, constituting 10%, served to fine-tune hyperparameters and prevent overfitting. The remaining 10% formed the testing set, a critical segment for evaluating the model's performance on unseen data.

Efficient training is essential for developing a proficient AI model. In this study, the collaborative capabilities of Google's platform were harnessed for their efficiency and cost-effectiveness. The model underwent training for a concise duration of one hour and 33 minutes. The collaborative nature of Google's platform facilitated seamless collaboration among researchers and expedited the training process, ensuring a cost-free and environmentally sustainable approach. The AI model utilized in this study was founded on a convolutional neural network (CNN) architecture, known as U-Net. This architecture, comprising encoder and decoder sections, has demonstrated efficacy in image segmentation tasks, making it well-suited for the intricacies of distinguishing skin lesions [18]. The model was trained for 50 epochs, each lasting approximately three seconds, totaling a training duration of around one minute and thirty seconds.

Performance evaluation encompassed key metrics, including accuracy, precision, recall, specificity, and F1 score. These metrics were all calculated using the confusion matrix. The area under the curve (AUC) was also considered, providing a comprehensive assessment of the model's discriminative power. These metrics collectively reflected the model's ability to accurately classify benign and malignant skin lesions. The selection of Kaggle.com as the source of the dataset, coupled with the strategic splitting of data and the utilization of Google's Collaboration platform, contributed to a robust methodology. This comprehensive approach aimed to ensure the model's effectiveness, generalizability, and relevance in real-world dermatopathological scenarios.

Ethical considerations

This study was deemed exempt from requiring Institutional Review Board approval as it solely relied on a publicly accessible dataset, without involving direct interaction with human participants. The dataset utilized in this research was obtained from openly accessible repositories, ensuring the complete safeguarding of personal information with respect to anonymity and confidentiality.

## Results

**Figure 1:**
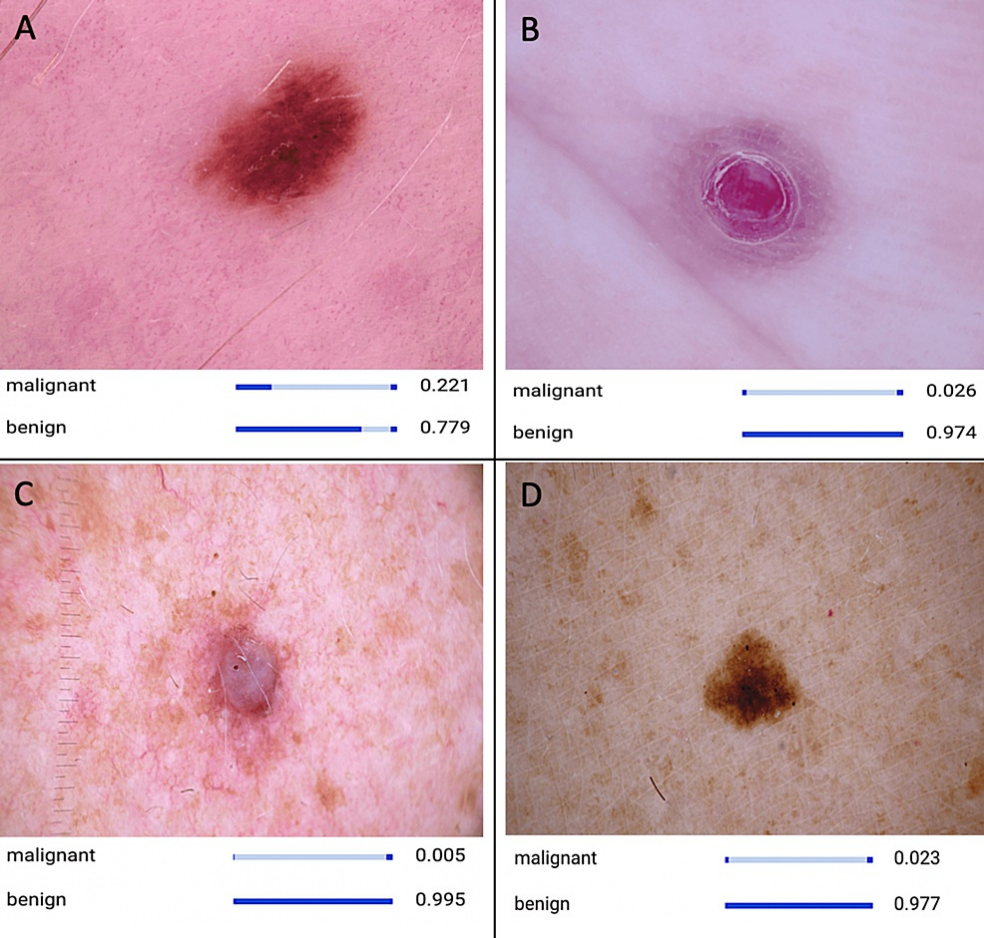
CNN model recognizing different images of benign lesions. (A) Nevus, (B) an ulcer, (C) dermatofibroma, and (D) a mole. The panel of images illustrates distinctive characteristics indicative of a benign skin lesion, such as symmetrical structure, consistent coloration, and scaly appearance. These features serve as crucial cues utilized by the developed AI model for detection and diagnosis. AI, artificial intelligence; CNN, convolutional neural network

**Figure 2:**
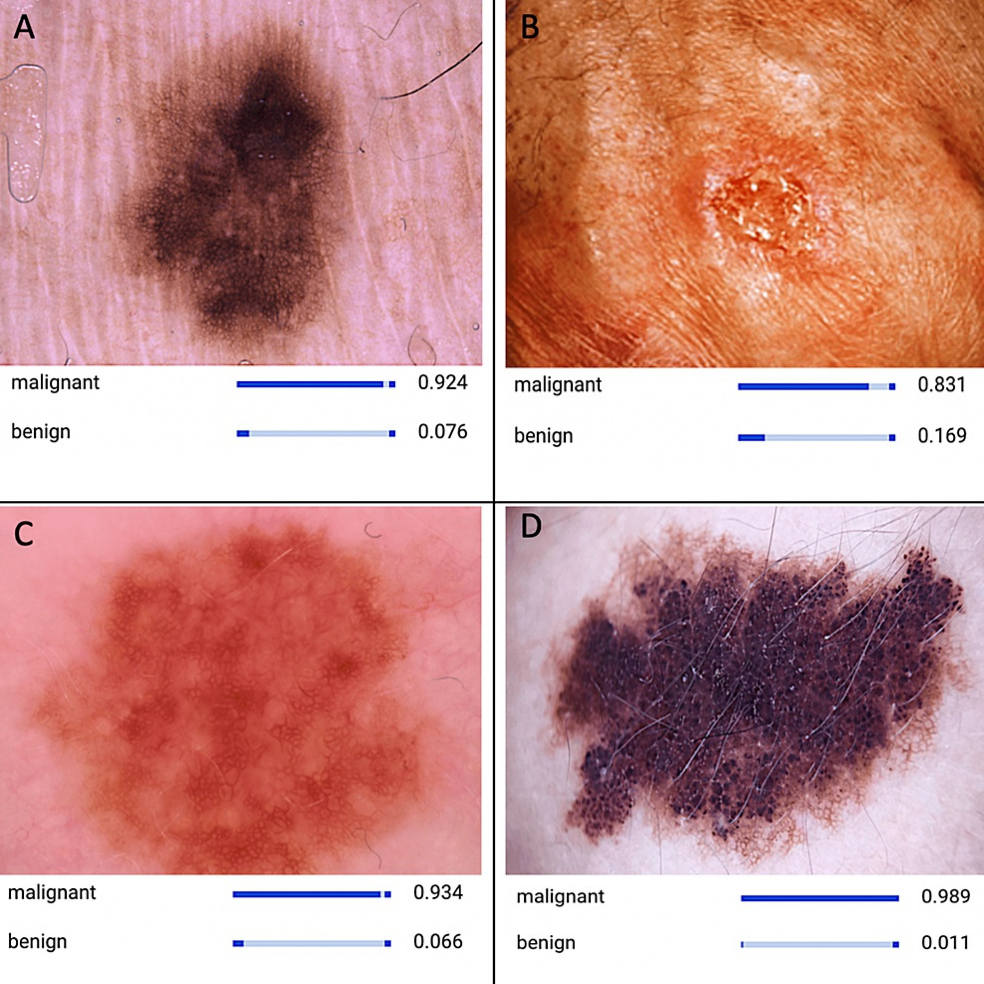
CNN model detecting images of different malignant lesions. (A) Melanoma, (B) squamous cell carcinoma, (C) basal cell carcinoma, and (D) Kaposi sarcoma. The board of images portrays distinctive features, suggesting a malignant skin lesion, which includes an asymmetrical shape, variation in color, and irregular borders. These characteristics serve as vital indicators leveraged by the developed AI model for the detection and diagnosis of malignant skin lesions. AI, artificial intelligence; CNN, convolutional neural network

In this study, we endeavored to develop an AI model capable of accurately discerning between pathology slides depicting benign and malignant skin lesions. The dataset that we used consisted of 500 images of benign skin lesions (Figure 1) and 500 images of malignant skin lesions (Figure 2). Leveraging Google's Collaboration platform, the model underwent expedited training, lasting a mere one hour and 33 minutes. The utilization of Google's servers ensured a cost-free and carbon-neutral training process, aligning with sustainability efforts in AI research.

**Figure 3:**
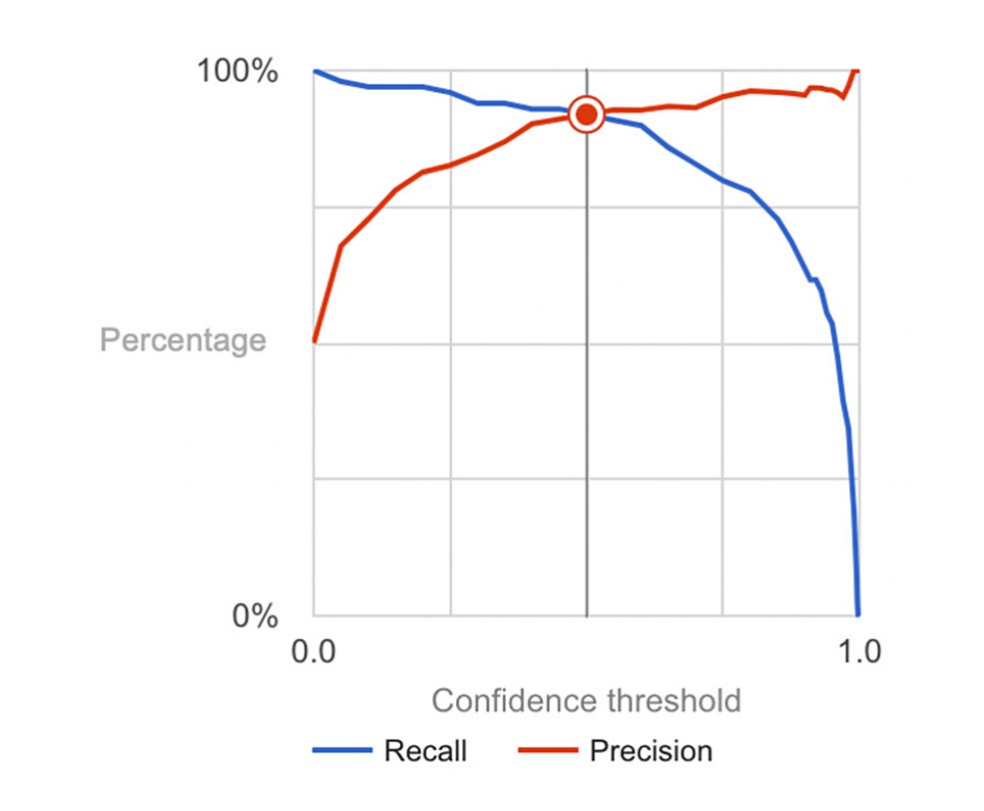
Precision-recall curve for benign and malignant lesion detection model. The graphical depiction illustrates the precision and recall of the neural network model across different confidence intervals.

**Figure 4:**
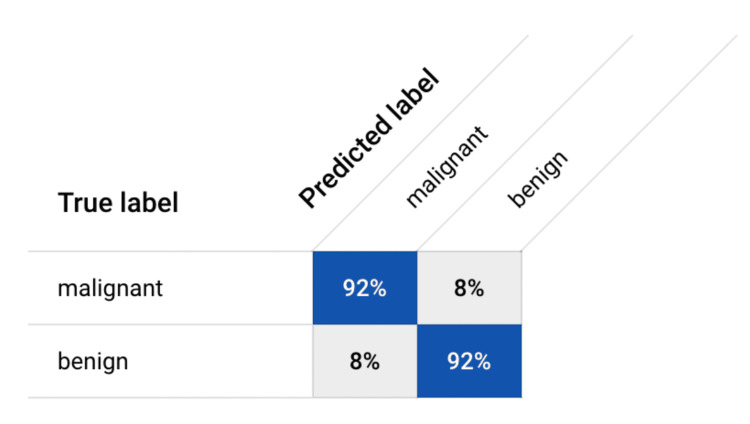
Confusion matrix. Various metrics, including accuracy, precision, recall (sensitivity), specificity, and F1 score, were computed using the data extracted from the confusion matrix.

Upon evaluation, our AI model exhibited robust performance metrics across various dimensions, affirming its efficacy in lesion classification. With an overall accuracy, precision, recall (sensitivity), specificity, and F1 score (calculated in Figure 3) all attaining an impressive 92%, the model demonstrated remarkable accuracy in distinguishing between benign and malignant lesions. These statistics were calculated using the confusion matrix (Figure 4). The model's AUC, which was determined by the crucial measure of its classification performance, was calculated to be 0.955, further highlighting its exceptional ability to accurately classify lesions. The precision value of 92% underscored the model's capability to correctly identify malignant lesions, while the equally high recall value of 92% emphasized its proficiency in capturing all positive cases of malignancy. Furthermore, the model showcased exceptional specificity of 92%, indicating its capacity to accurately identify benign lesions.

## Discussion

The findings presented in this study provide valuable insights into the transformative potential of AI within the domain of dermoscopy, particularly in the nuanced discrimination between benign and malignant skin lesions. Our meticulously developed AI model, trained on a rich and diverse dataset using Google's Collaboration platform, has demonstrated commendable performance metrics. These metrics include overall accuracy, precision, recall, specificity, and F1 score, all reaching an impressive 92%. The remarkable accuracy and balanced performance metrics of our AI model underscore its remarkable proficiency in effectively distinguishing between benign and malignant skin lesions. Notably, the model's consistent performance across multiple metrics highlights its robustness and reliability in handling complex classification tasks with precision and accuracy. The utilization of Google's Collaboration platform facilitated a swift and cost-effective training process, showcasing the transformative potential of cloud-based solutions in the development of AI technologies. This expedited training process not only accelerates the pace of AI model development but also highlights the scalability and accessibility of cloud-based AI platforms for researchers and developers worldwide.

When compared with previous research efforts focused on skin lesion classification, our AI model demonstrates competitive performance, as evidenced by the balanced performance metrics [12,19]. Similar to other investigations, our study has used publicly available datasets of dermatological images containing samples of benign and malignant skin lesions, sharing the common goal of training AI models to ensure robust performance [12]. Like other previous studies, our study assesses the performance of their AI models using standard evaluation metrics such as accuracy, precision, recall (sensitivity), specificity, and F1 score [12]. Our study distinguishes itself from previous studies in that our study demonstrates increased ability compared to previous studies that have used AI models to differentiate between different types of skin lesions [12]. For example, a study that aimed to differentiate between melanoma and levi had an accuracy of 88.6%, and a precision of 80.9%, while our model produced an accuracy of 92% and a precision of 92% [12]. Furthermore, our AI model attains a higher F1 score (92% compared to 88.3%), signifying a superior balance between precision and recall [12]. This validation of our model's effectiveness further underscores its potential to significantly enhance diagnostic accuracy in dermoscopy. The results obtained from the confusion matrix paint a promising picture of our model's capabilities, depicting its potential to substantially improve diagnostic accuracy and streamline clinical decision-making processes in dermatopathology. The combination of the expedited training process and the model's outstanding performance metrics emphasizes its relevance and applicability in real-world clinical settings, offering promising implications for enhanced patient care and the management of skin lesions. However, it is important to acknowledge the inherent variability in dataset composition, model architectures, and evaluation metrics across different studies. Further validation and refinement of our model may be necessary to optimize its performance and ensure its seamless integration into clinical workflows. Nonetheless, these findings represent a significant step forward in harnessing the power of AI to advance dermatopathological diagnostics and ultimately improve patient outcomes in dermatology.

While our study has yielded promising results, it is crucial to acknowledge and address several limitations that may impact the real-world application of our findings. First, the representativeness of the dataset utilized in our study may not fully encompass the diverse spectrum of benign and malignant skin lesions encountered in clinical practice. This limitation underscores the importance of expanding the dataset to include a broader range of lesion types, sizes, and clinical presentations. By incorporating data from various sources and populations, we can ensure the model's robustness and generalizability across different patient demographics and lesion characteristics. Additionally, variations in image quality and acquisition methods pose challenges to the model's real-world applicability. The utilization of low-quality images may compromise the reliability and effectiveness of the diagnostic process. Inaccurate or incomplete information derived from subpar images could potentially lead to misdiagnosis or delayed treatment, underscoring the importance of ensuring image quality standards are met to maximize the utility of AI in dermatology. Factors such as lighting conditions, camera settings, and imaging techniques can introduce variability in image appearance, potentially affecting the model's performance in practical settings. To address this challenge, future research efforts should focus on developing techniques to standardize image acquisition and enhance the model's robustness to variations in image quality Furthermore, while our study demonstrates the potential of AI in improving diagnostic accuracy in dermatopathology, validation studies conducted in clinical settings are essential to confirm the reliability and efficacy of the model in real-world scenarios. By evaluating the model's performance in diverse clinical environments and patient populations, we can assess its readiness for integration into routine dermatological workflows.

It is worth noting the additional time required to capture high-quality images during dermatological exams, as well as the potential investment in specialized technology. While integrating AI into dermatological practice may initially necessitate adjustments to workflow and investment in imaging equipment, the long-term benefits of AI-enabled diagnostics can outweigh these challenges. AI-driven image analysis algorithms can indeed expedite the interpretation of digital images captured during exams, compensating for the time invested in image acquisition. By automating lesion detection and classification, AI minimizes the manual effort required by dermatologists, allowing them to focus their expertise on areas that require further evaluation or intervention. Moreover, while specialized imaging equipment may be required for optimal image quality, advancements in smartphone technology have made it increasingly feasible to capture high-resolution images of skin lesions using handheld devices. This democratization of imaging technology extends the benefits of AI-enabled diagnostics beyond specialized dermatology clinics to primary care settings, where access to dermatologists may be limited. The value of AI in dermatology lies not only in its ability to expedite diagnostic processes but also in its potential to enhance diagnostic accuracy and consistency. AI-driven decision support systems can assist primary care providers in making informed decisions about patient management, reducing the risk of missed or delayed diagnoses. Ultimately, the benefits of AI in dermatology extend beyond time savings to encompass improvements in diagnostic accuracy, accessibility, and patient outcomes. By leveraging AI to augment clinical decision-making, dermatologists and primary care providers can deliver more efficient and effective care to patients, thereby optimizing the use of resources and improving overall healthcare delivery.

The successful development and integration of AI models with high accuracy in lesion classification have profound implications for modern medicine [20]. Early and accurate identification of malignant skin lesions, such as melanoma, is critical for improving patient outcomes by enabling timely intervention and treatment initiation [21]. By leveraging AI-powered diagnostic tools, healthcare providers can expedite the diagnostic process, leading to faster treatment decisions and improved patient care. AI accelerates dermatological diagnosis by optimizing lesion detection, classification, and decision-making. Through AI-powered image analysis, suspicious lesions are swiftly identified from digital images, streamlining biopsy prioritization. This automation reduces manual triaging time, expediting biopsies for potentially malignant lesions. Additionally, AI-driven decision support systems aid in post-biopsy decision-making by providing evidence-based treatment recommendations. By analyzing clinical data and biopsy findings, AI facilitates prompt referrals for further care based on AI-generated insights, improving diagnostic efficiency and patient outcomes in dermatology.

The seamless integration of AI into dermatology workflows holds promise for optimizing diagnostic processes and improving patient care [22]. By automating tasks such as lesion detection and classification, AI can streamline workflow efficiency and enhance diagnostic accuracy. However, challenges such as ensuring the interoperability of AI systems with existing healthcare infrastructure, addressing data privacy concerns, and mitigating potential biases in AI algorithms pose significant hurdles to successful integration. Despite these challenges, leveraging AI technologies in dermatology has the potential to revolutionize clinical practice, ultimately benefiting both healthcare providers and patients alike. By automating tasks such as lesion classification and triaging, AI presents an opportunity to revolutionize the efficiency of clinical decision-making processes. This automation not only expedites the diagnostic process but also frees up valuable time for dermatopathologists to allocate towards addressing more complex cases and delivering personalized patient care [23].

Traditional diagnostic methods often rely heavily on visual inspection by dermatologists, which can be subjective and prone to human error. AI-powered algorithms, on the other hand, can analyze digital images of skin lesions with remarkable precision and efficiency, aiding dermatologists in detecting subtle abnormalities and distinguishing between various skin conditions. While AI holds immense potential to revolutionize dermatological diagnostics, it is crucial to acknowledge that it is not infallible and may not always deliver accurate diagnoses. Despite this inherent limitation, AI remains invaluable in dermatology for several reasons. By analyzing vast datasets and leveraging sophisticated algorithms, these tools can provide clinicians with comprehensive insights into lesion characteristics, prognosis, and optimal therapeutic interventions. This enables clinicians to make well-informed decisions that are tailored to the individual needs and circumstances of each patient, ultimately leading to improved treatment outcomes and patient satisfaction [23]. Moreover, by automating routine tasks such as lesion detection and classification, AI frees up valuable time for dermatologists to focus on more complex cases and provide personalized patient care. Therefore, while AI may not be flawless, its potential to enhance diagnostic accuracy, improve workflow efficiency, and ultimately benefit patient outcomes underscores its continued value in dermatology. The inclusion of AI into dermatological workflows holds the potential to facilitate interdisciplinary collaboration and knowledge sharing among healthcare professionals [22]. Through the coordination of dermatopathologists, dermatologists, oncologists, and other specialists, AI fosters a collaborative approach to patient care that is centered around the holistic management of skin lesions and related conditions [23]. This interdisciplinary synergy not only enhances the quality of care delivered to patients but also promotes continuous learning and innovation within the field of dermatology [24].

Overall, the integration of AI-driven diagnostic tools into routine dermoscopy workflows represents a paradigm shift in the delivery of dermatological care by revolutionizing various aspects of clinical practice [22]. First, AI-driven diagnostic tools can augment dermatologists' capabilities by providing rapid and accurate assessments of skin conditions. By analyzing large datasets of clinical images and patient data, AI algorithms can assist in lesion detection, classification, and risk stratification, enabling dermatologists to make more informed diagnostic and treatment decisions. Moreover, AI can enhance the efficiency of dermatological workflows by automating repetitive tasks such as lesion triaging and documentation. By streamlining these processes, AI frees up valuable time for dermatologists to focus on more complex cases and provide personalized patient care. Additionally, AI-powered telemedicine platforms have the potential to expand access to dermatological care, particularly in underserved or remote areas. Through virtual consultations and remote monitoring, patients can receive timely dermatological evaluations and follow-up care without the need for in-person visits, thereby improving access to specialized care and reducing healthcare disparities.

By augmenting the capabilities of healthcare professionals and optimizing clinical decision-making processes, AI has the potential to revolutionize patient outcomes and pave the way for more efficient and effective dermatological practice [24]. By leveraging AI technologies, dermatology can transition towards a more data-driven, efficient, and patient-centered approach to care delivery, ultimately leading to improved access, quality, and outcomes in dermatological practice. In summary, while our study represents a significant step forward in harnessing the power of AI for dermoscopy, further research and validation are necessary to fully realize the potential of these technologies in modern medicine. By addressing the aforementioned limitations and advancing our understanding of AI's applicability in clinical practice, we can pave the way for improved patient outcomes and enhanced healthcare delivery in the field of dermatology [25].

## Conclusions

In conclusion, our study serves as an illuminating exploration into the potential and efficacy of AI within the realm of dermoscopy, focusing specifically on its adeptness in distinguishing between benign and malignant skin lesions. The AI model, meticulously developed through collaborative endeavors utilizing the advanced infrastructure of Google's platform, emerges as a testament to technological innovation in healthcare. Demonstrating remarkable performance, the model achieves an impressive 92% accuracy across a spectrum of critical metrics, further underscoring its competence and reliability in the nuanced task of differentiating between distinct types of skin lesions. This not only positions the model as a valuable tool in dermatological diagnostics but also highlights the transformative impact of AI in elevating precision and efficiency within medical decision-making processes.
